# Some Observations on the Barany Tests as Applied to Aviators

**Published:** 1918

**Authors:** 


**Some Observations on the Barany Tests as Applied to Aviators.** By H. L. BABCOCK. Abs. from the *Boston Med. and Surg. Jour.*, Dec. 13, 1917.

These tests determine the presence or absence of an intact and normally active static apparatus. The principle underlying them all is this : Movement of the endolymph in the semicircular canals in a given direction stimulates the sensitive hair cells in these canals, and produce definite phenomena. These are : 1) a twitching of the eyes, or nystagmus, of a certain type; 2) vertigo; 3) so-called " past-pointing "; 4) falling reactions.

The tests used for the aviation candidates are based on the turning reactions only, — the caloric and galvanic reactions being omitted entirely, — as the only object is to determine the integrity of the labyrinth and its tracts, and not differential diagnosis.

1. *Nystagmus.* The patient is seated in the Barany chair with head upright, but tilted 30 degrees forward and pressed back firmly against the head rest. With his eyes closed, he is turned to the right ten times in 20 seconds. The chair is then suddenly stopped and the candidate told to open his eyes and look straight ahead at some distant point. There should occur a horizontal nystagmus to the left of 26 seconds' duration. In a similar manner the candidate is turned to the left, although this is practically unnecessary, provided the first turning as given a normal reaction.

2. *Pointing tests.* The candidate, sitting in the chair, facing the examiner, closes his eyes and touches the examiner's finger, which is held in front of him. He next raises his arm to a perpendicular position, then lowers the arm, and attempts to find the examiner's finger — first with the right arm, then with the left. The normal individual is always able to find the finger. After turning the candidate to the right ten times in ten seconds, this test is repeated. During the last turn the stop pedal is released in order to lock the chair as it comes into position. This prevents any movement of the chair during the tests, which might offset the past-pointing. By past-pointing is meant the deviation of the candidate's finger to the right or left as he tries to locate that of the examiner. The right arm is tested, then the left, then the right, etc., until he ceases to past-point. In this instance the normal individual will past-point at least three times to the right with each arm. The eyes are kept closed throughout the test. The phenomenon of past-pointing is the result in this case of experimentally produced vertigo.

If the pointing tests are questionable, the vertigo is measured quantitatively in the following manner: The candidate is turned, with eyes closed, to the right at a uniform speed, and is asked to keep on telling the examiner in what direction he is being turned. Thus he says, "to the right, to the left," etc. After ten turns in ten seconds the chair is stopped and immediately he will say, "I am going to the left." The stop watch is started the same instant and kept running as long as the patient thinks he is going to the left. When he says, "I am standing still," the stop watch is stopped and the reading of the duration of the vertigo taken in seconds; in a normal case it is approximately 24 seconds. The test is repeated in a similar way by turning the patient to the left.

3. *Falling.* The candidate's head is inclined ninety degrees forward and held in place by a head rest. The eyes are closed. He is then turned to the right five times in ten seconds. On stopping the candidate raises his head and should fall to the right. As long as the head is kept in that position the vertigo remains horizontal, but on raising the head to an upright position, the vertigo at once becomes vertical, and the candidate, having the sensation of falling to the left, throws himself to the right in order to correct his supposed abnormal position. The test is then repeated turning to the left.

It is a well-known fact that stimulation of sensory nerve endings may affect the pulse-rate. Taking the general averages of 100 cases, pulse-rate findings immediately before and after turning show that the stimulation to the vestibular portion of the eighth cranial nerve end-organ by fifty revolutions, at varying rates of speed, produces an increase in the rate of heart action of nine beats per minute.

In a very few cases, an active reflex with the tenth cranial nerve has caused vomiting, especially after stimulation of the vertical canals. In no case has the candidate fainted.

